# Personalised Profiling of Innate Immune Memory Induced by Nano-Imaging Particles in Human Monocytes

**DOI:** 10.3389/fimmu.2021.692165

**Published:** 2021-08-06

**Authors:** Giacomo Della Camera, Mariusz Madej, Anna Maria Ferretti, Rita La Spina, Yang Li, Annunziata Corteggio, Tommaso Heinzl, Benjamin J. Swartzwelter, Gergö Sipos, Sabrina Gioria, Alessandro Ponti, Diana Boraschi, Paola Italiani

**Affiliations:** ^1^Institute of Biochemistry and Cell Biology (IBBC), National Research Council (CNR), Napoli, Italy; ^2^Istituto di Scienze e Tecnologie Chimiche “Giulio Natta” (SCITEC), National Research Council (CNR), Milano, Italy; ^3^European Commission, Joint Research Centre (JRC), Ispra, Italy; ^4^Stazione Zoologica Anton Dohrn, Napoli, Italy

**Keywords:** innate immunity, innate memory, nanoparticles, imaging materials, monocytes

## Abstract

Engineered nanoparticles used for medical purposes must meet stringent safety criteria, which include immunosafety, *i.e.*, the inability to activate possibly detrimental immune/inflammatory effects. Even medical nanomaterials devoid of direct immunotoxic or inflammatory effects may have an impact on human health if able to modify innate memory, which is the ability to “prime” future immune responses towards a different, possibly more detrimental reactivity. Although innate memory is usually protective, anomalous innate memory responses may be at the basis of immune pathologies. In this study, we have examined the ability of two nanomaterials commonly used for diagnostic imaging purposes, gold and iron oxide nanoparticles, to induce or modulate innate memory, using an *in vitro* model based on human primary monocytes. Monocytes were exposed in culture to nanoparticles alone or together with the bacterial agent LPS (priming phase/primary response), then rested for six days (extinction phase), and eventually challenged with LPS (memory/secondary response). The memory response to the LPS challenge was measured as changes in the production of inflammatory (TNFα, IL-6) and anti-inflammatory cytokines (IL-10, IL-1Ra), as compared to unprimed monocytes. The results show that both types of nanoparticles can have an effect in the induction of memory, with changes observed in the cytokine production. By comparing nanomaterials of different shapes (spherical *vs*. rod-shaped gold particles) and different size (17 *vs*. 22 nm diameter spherical iron oxide particles), it was evident that innate memory could be differentially induced and modulated depending on size, shape and chemical composition. However, the main finding was that the innate memory effect of the particles was strongly donor-dependent, with monocytes from each donor showing a distinct memory profile upon priming with the same particles, thereby making impossible to draw general conclusions on the particle effects. Thus, in order to predict the effect of imaging nanoparticles on the innate memory of patients, a personalised profiling would be required, able to take in consideration the peculiarities of the individual innate immune reactivity.

## Introduction

Among the several biomedical applications of nanomaterials, nanoparticles (NP) of various composition and characteristics represent contrast agents of new generation, with improved sensitivity and imaging capacity (1). Medical imaging technologies in which NP are applied go from fluorescence imaging to positron emission tomography, and include magnetic resonance imaging, ultrasound, computed tomography and single-photon emission computed tomography (2). Among nanoimaging materials, iron oxide and gold NP are widely used for a number of reasons: they are easy to synthesize and have high colloidal stability, can be easily functionalized thereby acquiring new desired surface characteristics, and are safe and biocompatible, as proven by many years of use in different diagnostic and therapeutic approaches (3–5).

Iron oxide nanoparticles (FeOxNP) have been long applied in both therapeutic and diagnostic approaches (6). Therapeutically, they are used in treatment of anaemia (7) and in magnetic fluid hyperthermia (8). In diagnostic imaging, FeOxNP are applied in magnetic resonance angiography (9–11) and imaging of organs/tissues such as liver (12), lymph nodes (13–15) and bone marrow (16), and imaging in pathological situations, *e.g.*, tumour perfusion (17–19), atherosclerotic plaques and thrombosis (20–22) and various types of inflammation (23–25). Importantly, FeOxNP are useful for identifying macrophages in atherosclerotic plaques (26) and in other organs/tissues, and they have been reported able to polarize macrophages towards an anti-tumoral phenotype (27, 28).

Gold nanoparticles (AuNP) are also used in several imaging approaches, in particular X-ray imaging and computed tomography (29), exploiting the high gold atomic number that allows for a higher X-ray absorption efficiency compared to other agents and tissues (bone, soft tissues) (2, 30). They are also applied in other approaches, such as the photoacoustic imaging of brain vasculature (31). AuNP are considered safe and biocompatible, are easy to produce in various sizes and shapes, and can be surface-modified to display different functionalities. AuNP can act as cell tracers, once taken up by cells (29), and therefore they have been used for monitoring tumour growth (32), to image blood flow (33) and to visualise monocyte migration into atherosclerotic plaques (34).

Despite their wide range of applications, our understanding of the biological interactions, toxicity, uptake and clearance routes of these NP is still incomplete, in particular regarding their long-term interaction with the immune defensive system. By focusing in particular on inflammation/innate immunity, the effects of FeOxNP are variable and likely depending on type/shape/size of particles, concentration, route of administration and target immune cells (35). As already mentioned, FeOxNP were shown able to drive intratumor macrophage polarization towards the M1 inflammatory and tumoricidal phenotype (28), supporting their ability to induce innate/inflammatory activation, as shown *in vitro* by the release of inflammatory cytokines (IL-1β, IL-12, TNFα and IFN-γ) from macrophages and immature dendritic cells (36, 37). Several studies report the capacity of FeOxNP to induce the production of inflammatory/toxic reactive oxygen species [reviewed in (35, 38)]. Other studies, however, do not find inflammation-related effects on macrophages, dendritic cells and microglial cells *in vitro* (36, 39–42). While AuNP are generally considered biocompatible, variable results have been obtained, depending on the experimental model/cell type used, the dose, size and shape of the NP (43–46). On immune cells such as monocytes, macrophages and dendritic cells, AuNP in general show limited or no immunotoxic/inflammatory effect *in vitro* [(43, 44), reviewed in (47, 48)]. An important issue, when examining the inflammatory effects of NP on immune cells *in vitro*, in particular on human cells, is the possible presence of contaminating endotoxin, a ubiquitous bacterial product with high inflammatory activity present also in conditions of microbiological sterility. The presence of endotoxin on the surface of many NP types can confer to the NP a potent inflammatory activity that is undetectable in clean NP (49–52).

In this study, we have examined the capacity of AuNP and FeOxNP to interact with cells of the innate immune system and induce their inflammatory activation (53–55). To increase the reliability of results, we have selected an *in vitro* model based on primary human cells (blood monocytes), thereby avoiding the unrealistic experimental systems based on non-human or transformed/tumour cell lines, and we have used particles that were endotoxin-free at the concentrations used. The use of two different shapes for AuNP (spheres and rods) and two different sizes for FeOxNP (17 and 22 nm) would allow for detecting the capacity of human innate immune cells to distinguish between chemistry, shape and size of the interacting particles.

Besides a direct effector function based on non-specific inflammation-related activities (production of inflammatory factors, prostaglandins and other lipid mediators, reactive oxygen and nitrogen species, lytic enzymes, etc.), innate immune cells have the capacity of developing “memory”. Innate immune memory is a phenomenon known since several decades, which has been recently re-discovered under the name of trained immunity (56–61). Similar to the well-known adaptive immune memory at the basis of vaccine efficacy, innate memory implies that innate cells exposed to a stimulus can retain the memory of such exposure and react to a subsequent stimulation differently, compared to cells that had not been previously exposed. This optimized secondary “memory” response could be stronger (potentiation, trained immunity) or weaker (tolerance) compared to the primary response, in order to attain the best protection against infectious/stressful challenges and at the same time preserve the structural and functional integrity of the affected tissues/organs. At variance with adaptive immune memory, innate memory is at least partially non-specific, meaning that the memory reaction is independent of the challenge, *i.e.*, the optimized memory reaction occurs also in response to a challenge that is different from the memory-inducing “priming” stimulus.

Few studies have examined the capacity of NP to induce innate memory. In mouse macrophages, pristine graphene could induce a memory profile that resulted in an increased innate/inflammatory response to subsequent stimulations (62). In human monocytes and macrophages, AuNP could induce memory responses either directly, by priming cells for a different secondary response, or by modulating the memory-inducing capacity of strong stimuli such as LPS (which induces tolerance) and BCG (the *Mycobacterium bovis* strain used as tuberculosis vaccine, which induces a potentiated secondary responses) (63–67). Thus, in this study we have focused on the ability of FeOxNP and AuNP of different sizes and shapes not only to induce a primary innate response (measured as balance between the induction of inflammatory *vs*. anti-inflammatory cytokines) but in particular to induce and/or modulate innate memory.

## Materials and Methods

### Nanoparticle Synthesis and Characterisation

Rod-shaped AuNP (AuNP ROD) were purchased by Nanopartz Inc. (cat. A12-5-780-CIT-DIH-1-100-CS-EP, lot. J7251; Nanopartz Inc., Loveland, CO, USA). Declared characteristics were: capping with citrate (3 mM), diameter 5 nm, length 18 nm, ζ-potential -35 mV, concentration 39 µg/mL in water, pH 7.0, sterile and endotoxin-free. Particles were re-characterised before use in the biological experiments. The company refused to release information on the synthesis procedure.

Spherical AuNP (AuNP SPH) were prepared using the procedure of Turkevich et al. (68), with slight modifications. All reagents and solutions were prepared in LAL Reagent Water (LRW; Associates of Cape Code, Inc., East Falmouth, MA, USA), to ensure endotoxin-free conditions. Briefly, 5 mL of 0.01 M tetrachloroauric acid tri-hydrate (HAuCl4·3H2O; Sigma-Aldrich, Merck KGaA, St. Louis, MO, USA) were diluted in 90 mL water. The solution was rapidly heated to 97°C in a microwave heating system (Discover S; CEM Corporation, Matthews, NC, USA), using a maximum power of 250 W, and allowed to equilibrate for 5 min under constant mechanical stirring. The reduction reaction was started by injecting 2.5 mL 0.1 M trisodium citrate di-hydrate (Sigma-Aldrich) into the gold solution, in order to reach a 2.5 mM final citrate concentration. The reaction mixture was kept at 97°C for 20 min under stirring, then the reaction vessel was rapidly cooled on ice, and the NP solution stored at +4°C until use.

**Figure 1:**
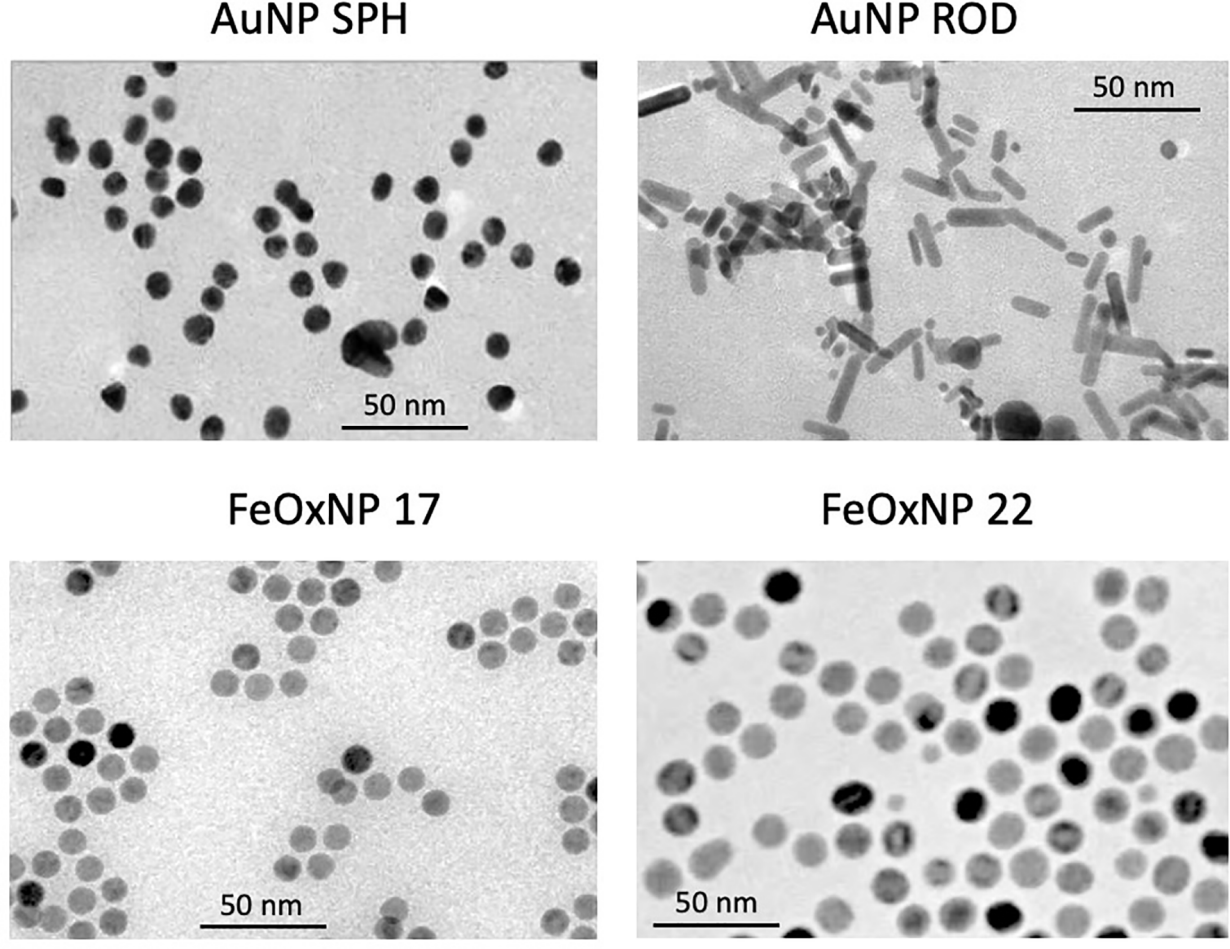
Size and morphology of the AuNP and FeOxNP used in this study. TEM images of spherical AuNP (upper left panel), rod-shaped AuNP (upper right panel), FeOxNP 17 (lower left panel) and FeOxNP 22 (lower right panel). Size bars (50 nm) are shown in each panel.

Details of the synthesis and characterisation of FeOxNP coated with zwitterionic dopamine sulfonate (ZDS) have been published elsewhere (42). For all NP types, the main physico-chemical characteristics are reported in **Figure 1** and **Tables 1**, **2**.

**Table 1:** NP morphology.

Parameter	AuNP SPH	AuNP ROD^a^	FeOxNP 17	FeOxNP 22
*<d>*(nm)	13.0	20.0, 6.5	16.8	22.4
σ_d_ (nm)	0.8	13.0, 0.8	0.9	1.5
σ_d_/*<d>*	6%	7%, 12%	5%	7%

The mean diameter <d> is reported along with the standard deviation of the diameter σ_d_ and the coefficient of variation σ_d_/<d>.

a Length, width.

**Table 2:** NP hydrodynamic features.

Parameter	AuNP SPH	AuNP ROD	FeOxNP 17	FeOxNP 22
*D*v (nm)^a^	16 ± 2 (WFI)^b^	17 ± 4 (WFI)	20 ± 1 (WFI)	21 ± 3 (WFI)
	17 ± 7 (PBS 1x)	22 ± 4 (PBS 1x)	NA	NA
	100 ± 20 (RPMI)	120-170 (RPMI)	18 ± 2 (RPMI)^d^	17 ± 5 (RPMI)^d^
	38 ± 2 (RPMI)^c^	22 ± 2 (RPMI)^c^	NA	NA
ζ-potential (mV)^e^	−53 ± 2	−39 ± 4	−18 ± 2	−29 ± 3
Endotoxin activity	0.45 EU/mg	30.8 EU/mg	58.3 EU/mg	<16 EU/mg

a Volume-weighted hydrodynamic diameter; for Au nanorods, it is the volume-weighted hydrodynamic diameter of spherical NP having the same average diffusion coefficient of the nanorods.

b WFI, water for injection.

c NP were pre-treated with 50% human serum.

d RPMI plus 5% FBS.

e in PBS 1x.

NA, not available.

All NP were pre-coated with human AB serum (Sigma-Aldrich) immediately before use in monocyte stimulation experiments. Briefly, NP suspensions were admixed 1:1 with heat-inactivated serum and incubated for 2 h at 37°C in an orbital shaker at 500 rpm. NP were then diluted in culture medium to the concentration required for use in the biological assays, and serum concentration was adjusted to 5%. As already described elsewhere (52), coating with serum significantly increased the colloidal stability of AuNP (data not shown).

Transmission electron microscopy (TEM) images of untreated NP were recorded using a JEOL-JEM 2100 microscope operated in transmission mode at an acceleration voltage of 120 kV. TEM specimens were prepared by depositing 3 μL of non-treated NP dispersion onto Formvar carbon-coated grids (S162 Formvar/Carbon, 200 mesh copper grids; Agar Scientific Ltd, Stansted, UK) and left to dry at room temperature in a desiccator (overnight) and under vacuum for 2 h. TEM images from different regions of the grid were analysed using the ImageJ Software (http://rsb.info.nih.gov/ij/). NP size distributions and shapes were evaluated using the Particle Size Analyzer imageJ macro or by manual processing. More than 350 isolated primary NP were assessed for each sample.

Dynamic light scattering (DLS) experiments were carried out using a Malvern Zetasizer Nano-ZS instrument (Malvern Panalytical Ltd, Malvern, UK) equipped with a light source of 632.8 nm wavelength at a fixed scattering angle of 173°. Data were analysed by the CONTIN software (69, 70). The ζ-potential was measured by electrophoretic light scattering (ELS), using the same instrumentation. For each NP suspension, dilutions were prepared in water for injection (WFI), PBS, and RPMI. Then, 0.6 mL of each sample dilution were transferred to a disposable cuvette (cat. ZE0040; BrandTech Scientific, Inc., Essex, CT, USA) for DLS measurement, whereas 1.2 mL was loaded in a disposable folded capillary cell (cat. DTS1070, Malvern) for ELS measurement. Each sample was measured twice at 25°C, after an equilibration step of 120 sec with an acquisition time of 80 sec. The hydrodynamic diameter was reported as the volume-weighted mean diameter (*D_V_*).

### Human Monocyte Isolation and Differentiation of Monocyte-Derived Macrophages

Blood was obtained from healthy donors, upon informed consent and in agreement with the Declaration of Helsinki. The protocol was approved by the Regional Ethics Committee for Clinical Experimentation of the Tuscany Region (Ethics Committee Register n. 14,914 of May 16, 2019). Monocytes were isolated by CD14 positive selection with magnetic microbeads (Miltenyi Biotec, Bergisch Gladbach, Germany) from peripheral blood mononuclear cells (PBMC), obtained by Ficoll-Paque gradient density separation (GE Healthcare, Bio-Sciences AB, Uppsala, Sweden). Monocyte preparations used in the experiments were > 95% viable and > 95% pure (assessed by trypan blue exclusion and cytosmears). Monocytes isolated with this method were not activated, based on analysis of expression of inflammation-related genes (*IL1B*, *TNFA*) and release of the encoded proteins, compared to monocytes within PBMC (*i.e.*, after Ficoll-Paque separation) and whole blood (*i.e.*, after withdrawal with anticoagulants) (data not shown).

Monocytes were cultured in culture medium (RPMI 1640 + Glutamax-I; GIBCO by Life Technologies, Paisley, UK) supplemented with 50 µg/mL gentamicin sulfate (GIBCO) and 5% heat-inactivated human AB serum (Sigma-Aldrich). Cells (5x10^5^) were seeded in a final volume of 1.0 mL in wells of 24-well flat bottom plates (Corning^®^ Costar^®^; Corning Inc. Life Sciences, Oneonta, NY, USA) at 37°C in moist air with 5% CO_2_. Monocyte stimulation was performed after overnight resting.

### Human Monocyte Activation and Induction of Innate Memory

For assessing the primary response to stimulation, monocytes were exposed for 24 h to culture medium alone (medium/negative control) or containing 1 ng/mL LPS (positive control; from *E. coli* O55:B5; Sigma-Aldrich), serum pre-coated NP, or LPS in the presence of NP.

For memory experiments, after the first exposure to stimuli for 24 h and supernatant collection, cells were washed and cultured with fresh culture medium for 7 additional days (one medium change after 4 days). During this period, the activation induced by previous stimulation subsides, and cells return to their baseline status (as determined by evaluation of inflammation-related cytokines in the supernatant). After this resting phase, the supernatant was collected, and cells were challenged for 24 h with fresh medium alone or containing 5 ng/mL LPS (a 5x higher concentration than in the primary stimulation).

All supernatants (after the first stimulation, after the resting phase and after the challenge phase) were frozen at −20°C for subsequent cytokine analysis. By visual inspection, cell viability and cell number did not substantially change in response to the different treatments.

### Cytotoxicity Evaluation

The direct toxicity of NP on monocytes was tested as release of lactate dehydogenase (LDH). Briefly, monocytes (1.2x105 cells/well of 96-well plates; Corning Inc.) were incubated for 24 h in 200 µL culture medium alone or containing serum-coated NP in triplicate. Positive control wells received 200 µL of 0.1% Triton-X 100. At the end of the incubation, release of the cytoplasmic enzyme LDH was measured in the supernatant using a colorimetric assay (LDH-Cytotoxicity Colorimetric Assay Kit; BioVision, Inc., Milpitas, CA, USA).

### Assessment of Endotoxin Contamination

Endotoxin contamination in NP was assessed with a commercial chromogenic LAL assay (Pyros Kinetix® Flex (Associates of Cape Cod, Inc.), following a protocol adapted for use with particulate agents (71). As the assay readout is paranitroaniline (pNA) at 405 nm, preliminary controls were run to assess the possible interference of NP (direct optical reading at 405 nm and interference with detection of different concentrations of synthetic pNA). NP were used in the LAL assay at concentrations that did not interfere with the assay readout. An additional control was run, to assess the possible interference of NP with the assay components/reagents, which consisted in spiking the NP samples with a known amount of LPS (0.5 EU/mL) and assessing the recovery of spiked endotoxin. A recovery rate between 80 and 120% was considered acceptable. The endotoxin contamination was therefore reliably assessed at NP dilutions that did not interfere with the 405 nm readout and in which 80-120% spiked endotoxin could be recovered. The LAL assay was run with Glucashield® (Associates of Cape Cod, Inc.), to eliminate possible false positives due to the presence of glucans, using a dedicated tube reader and software (Associates of Cape Cod, Inc.). The assay sensitivity was 0.001 EU/mL.

### Transmission Electron Microscopy for NP Uptake

Cells (1x10^6^ in wells of 6-well flat bottom plates) were exposed to AuNP for 24 h, then fixed, stained and embedded in EPON resin for sectioning (EM UC7 ultramicrotome; Leica Microsystems, Wetzlar, Germany) (65). TEM images were obtained using a Tecnai 12 transmission electron microscope (FEI Company, Hillsboro, OR, USA).

### Cytokine Analysis

The levels of the human inflammatory cytokines TNFα and IL-6 and of the anti-inflammatory factors IL-10 and IL-1Ra were assessed by ELISA (R&D Systems), using a Cytation 3 imaging multi-mode reader (BioTek, Winooski, VT, USA).

### Statistical Analysis

Data were analyzed using the GraphPad Prism6.01 software (GraphPad Inc., La Jolla, CA, USA). For cytokine production, results are presented as ng produced cytokine/10^6^ plated monocytes. Results are reported as mean ± SD of values from 2-6 replicate samples from the same donor. Statistical significance of differences is indicated by *p* values, calculated using unpaired and two-tailed Student’s *t*-test (**Figures 4**, **5**).

## Results and Discussion

### Physico-Chemical Characterisation of the NP Used in This Study

Two types of nano-imaging materials have been evaluated in this study, gold and iron oxide (Fe_3−_ *_x_*O_4−_ *_x_*, 0 ≤ *x* ≤ 1, cubic ferrite structure). Gold (Au) NP of two different shapes have been assessed, quasi-spheres with a diameter of 13 nm (surface area 531 nm^2^), and rods of 20 x 6.5 nm (surface area 408 nm^2^). Both AuNP types are stabilized by citrate. Spherical iron oxide (FeOx) NP of different size were used, 16.8 and 22.4 nm diameter (the latter having a surface area almost double *vs*. the former, 1576 *vs*. 887 nm^2^), both stabilized by zwitterionic dopamine sulfonate (ZDS). The morphology of the inorganic core of AuNP and FeOxNP is depicted in the **Figure 1** (TEM images), and their morphological characteristics summarised in **Table 1**. The corresponding size histograms are reported in **Supplementary Figure 1**. All NP are monodispersed, with size varying by less than (or close to) 10% from the average value.

The hydrodynamic features of the AuNP and FeOxNP are reported in **Table 2**, in which their endotoxin contamination is also shown. AuNP and FeOxNP are well-dispersed and colloidally stable in water, and their volume-weighted hydrodynamic diameter (*D_V_*) is consistent with the TEM-derived core size <*d*>. Both types of AuNP are well-dispersed also in PBS 1x, with similar *D_V_*, but they aggregate in RPMI-1640 medium, as shown by the strong increase in *D_V_*. When AuNP are pre-treated with human serum before dispersion in RPMI medium, they have *D_V_* close to those they display in WFI and PBS. Both types of FeOxNP do not aggregate for at least 6 h in RPMI-1640 medium added with 5% foetal bovine serum (FBS). Furthermore, their *D_V_* shows that they do not adsorb proteins.

The colloidal stability of AuNP and FeOxNP is due to their negative ζ-potential that ensures electrostatic repulsion between NP. The different stability of AuNP *vs*. FeOxNP is due to their coating. The citrate anions are weakly bound (physisorbed) to the AuNPs, and are thus not able to prevent aggregation in RPMI-1640 medium, probably because they are displaced by the non-salt components in the culture medium. Conversely, the zwitterionic sulfobetaine ZDS is strongly bonded to the FeOxNP and is able to protect NP from aggregation and protein adsorption (42), thanks to its hydrophilicity and overall neutrality. The pre-treatment with human serum afforded colloidal stability in culture medium for spherical AuNP and, up to a concentration of 1.4 µg/mL, also for rod-shaped AuNP. The similarity of the *D_V_* between untreated AuNP in WFI and PBS and serum-coated AuNP in RPMI-1640 medium suggests that the latter are stabilized by a protein coating. However, the position of the major peak of the intensity-weighted size distribution of AuNP SPH increases by about 6 nm when they are pre-treated with human serum, suggestive of the formation of a biocorona on the NP surface (**Supplementary Figure 2**). The major serum component within the NP biocorona was a 60 kDa protein, most likely albumin (data not shown).

### Selection of the NP Concentrations for Biological Studies

The NP concentrations to be used in biological experiments were selected based on two criteria, *i.e.*, the endotoxin contamination (in order to use of the highest possible concentration with endotoxin contamination not exceeding 0.1 EU/mL, *i.e.*, the concentration at which there is little/no human monocyte activation *in vitro*; 45) and the surface area (in order to use NP concentrations comparable by total surface area). The latter criterion is based on the fact that NP are incubated with human serum before being added to human monocytes in culture, to reproduce the *in vivo* conditions in which NP are exposed to biological fluids and expected to form a biomolecular coating of adsorbed proteins/molecules on their surface, which may change the mode of their interaction with living cells. Thus, a comparable NP surface area may allow for a more comparable interaction with microenvironmental molecules (the biocorona) and with cells in the experimental system. We have calculated a surface area of 27 mm^2^ as the optimal dose to compare the different NP types, which corresponds to 5.7 µg AuNP SPH (with an endotoxin contamination of 0.003 EU), 2 µg FeOxNP 17 (endotoxin 0.117 EU) and 2.7 µg FeOxNP 22 (endotoxin <0.043 EU), the dose-limiting factor being the endotoxin contamination of FeOxNP 17. For AuNP ROD, a 2.7x lower dose was used (surface area 10 nm^2^, 1.4 µg NP, endotoxin 0.043 EU), because of problems of aggregation at higher concentrations. Thus, the NP concentrations used in culture were 5.7 µg/mL for AuNP SPH, 1.4 µg/mL for AuNP ROD, 2.0 µg/mL for FeOxNP 17 and 2.7 µg/mL for FeOxNP 22.

### Primary Response of Human Monocytes to NP Exposure

NP aggregation during *in vitro* assays is a common disturbing factor when studying NP-cell interactions. In our case, aggregation is a very minor factor since (i) both AuNP types are colloidally stabilized by the pre-treatment with human serum (likely due to protein adsorption), and (ii) both FeOxNP types are coated with ZDS, which is known to endow high colloidal stability (42). Therefore, we hold that NP aggregation is of minor importance in the present study, in particular at the selected concentrations.

Before assessing the effects of the selected NP on innate/inflammatory responses of human monocytes, their possible direct toxicity was examined. As already mentioned, all NP were pre-coated with human AB serum (see *Materials and Methods*).

Monocytes isolated from blood of individual donors were exposed to NP at the established concentrations for 24 h. Cell death was assessed in terms of release of the cytoplasmic enzyme LDH. No toxicity was detected for any of the NP type on monocytes of any donor (data not shown). Uptake of NP by monocytes was monitored visually. Cells took up the NP abundantly, as expected for professional phagocytes, and stored them within vesicles (see examples for AuNP SPH in **Supplementary Figure 3**). Since NP were not toxic for monocytes, there was no correlation between the NP uptake and the NP toxicity.

The primary response of human monocytes to NP was assessed after exposure in culture for 24 h. Monocytes are cells of the innate immune system, involved in immediate defensive reactions that include inflammatory responses. Thus, monocyte activation was evaluated in terms of production of four innate cytokines, the inflammatory factors TNFα and IL-6, and the anti-inflammatory cytokines IL-10 and IL-1Ra. As positive control, cells were exposed to LPS, which is an excellent activator of human monocyte innate/inflammatory responses. Cells were also co-exposed to LPS and NP, in order to detect possible NP-dependent modulation of LPS-induced activation. For this reason, a sub-optimal LPS concentration was selected (1 ng/mL), in order to allow us to detect both positive and negative NP effects.

**Figure 2:**
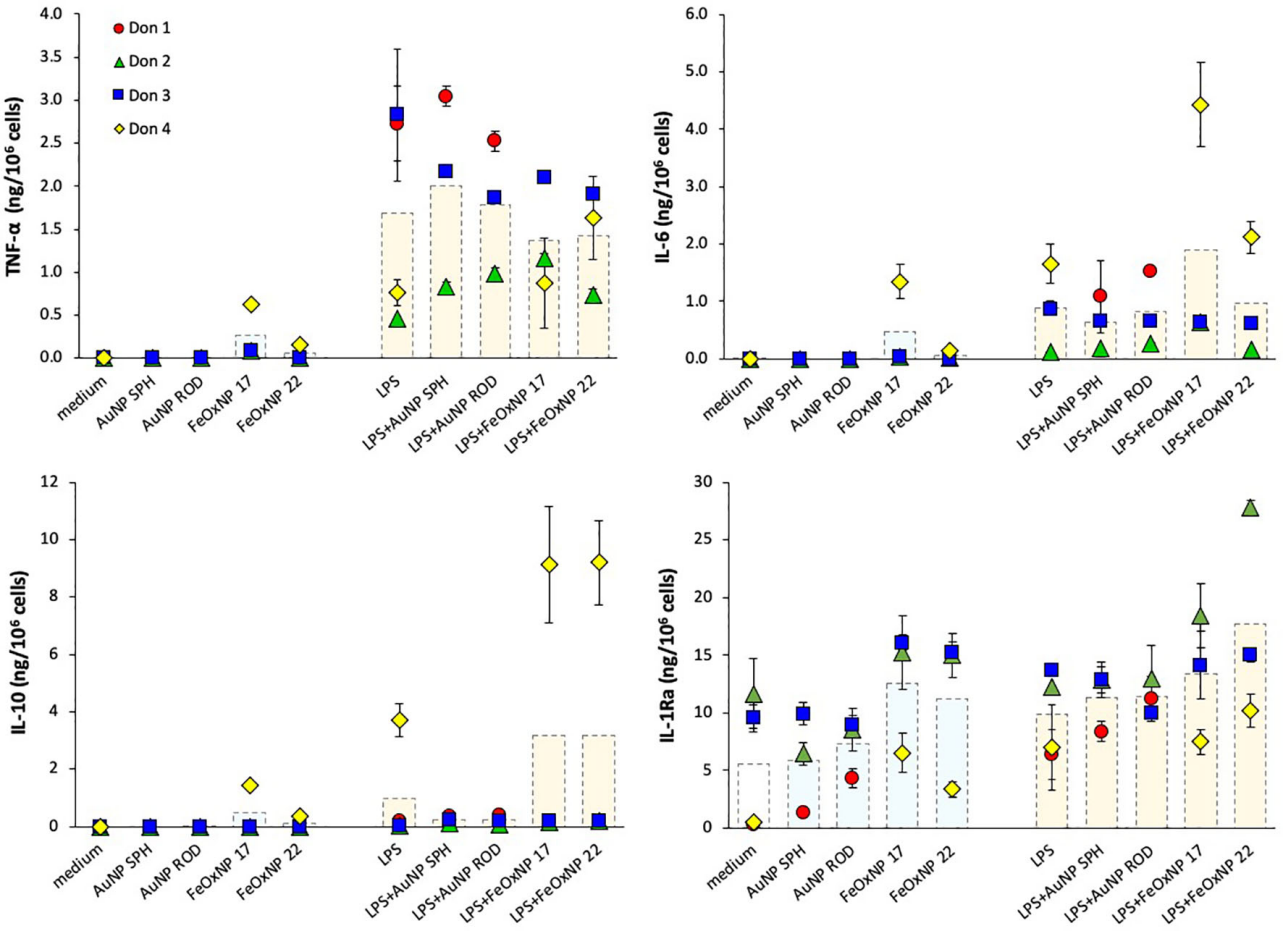
Innate immune primary response of human monocytes to NP alone or upon co-exposure with LPS. Human monocytes isolated from blood of four individual donors (red, green, blue, and yellow symbols) were cultured for 24 h in culture medium alone (empty columns) or containing serum-precoated NP: 5.7 µg/ml spherical AuNP (AuNP SPH), 1.4 µg/ml rod-shaped AuNP (AuNP ROD), 2 µg/ml spherical FeOxNP of 16.8 nm diameter (FeOxNP 17) and 2.7 µg/ml spherical FeOxNP of 22.4 nm diameter (FeOxNP 22) alone (light blue columns) or together with 1 ng/ml LPS (orange columns). The production of TNFα (upper left), IL-6 (upper right), IL-10 (lower left) and IL-1Ra (lower right) was measured in the 24 h supernatants by ELISA. Data are presented as individual donors’ values (coloured symbols) ± SD and as mean of the individual values (dotted columns).

In line with previous evidence, none of the NP could induce a measurable reactivity in human monocytes, being inactive in inducing inflammatory cytokines (**Figure 2**, upper panels), apart from a measurable induction with FeOxNP 17 in one donor. When examining anti-inflammatory cytokines, it should be noted that, similar to inflammatory factors, no detectable IL-10 levels were produced in baseline unstimulated conditions, whereas the baseline levels of IL-1Ra were measurable (**Figure 2**, lower panels). Neither AuNP nor FeOxNP could stimulate monocytes to produce IL-10 (again with the exception of FeOxNP 17 for one donor), whereas a tendency to increase in IL-1Ra production was detected in response to FeOxNP in all donors (while AuNP are essentially inactive, regardless of shape). LPS significantly activated the production of inflammatory factors in monocytes, although with a substantial donor-to-donor variability. Such variability was also evident in the ability of NP to modulate the response to LPS, with donors showing a NP-dependent amplification of the response to LPS, others showing a decrease and others no substantial changes. In the case of the anti-inflammatory cytokine IL-10, it should be noted that, as expected, its induction by the selected LPS concentration was null, except for one donor. Only in this donor, FeOxNP seemed able to directly induce detectable IL-10 levels, as well as to increase LPS-induced IL-10 production. In the case of the other anti-inflammatory factor, IL-1Ra, the significant baseline production was variably affected by NP and only partly increased by LPS (in 2/4 donors). Co-exposure to LPS and NP did not substantially affect IL-1Ra production, although with some donor-to-donor variability, as in the case of the high IL-1Ra increase in response to LPS plus FeOxNP 22 in one donor. The donor-dependent NP activities, already observed in previous studies, suggest the need for a personalised evaluation of NP effects when safety assessment is required, *e.g.*, before their clinical diagnostic use.

### Memory Response of Human Monocytes Exposed to NP

After exposure for 24 h to NP, LPS or their mixture, cells were washed (to eliminate the priming agents) and cultured for 7 days in fresh culture medium to allow return to baseline. The culture medium was refreshed after 4 days. The extinction of cell activation was confirmed by examining the production of cytokines released in the culture medium at the end of the resting period (representing the cytokine release in the last 3 days of resting) (data not shown). After the extinction period, cells were either exposed to medium alone or challenged with 5x higher LPS concentration, relative to priming, in agreement with the concept that memory can shape the host capacity to react to more severe challenges.

**Figure 3:**
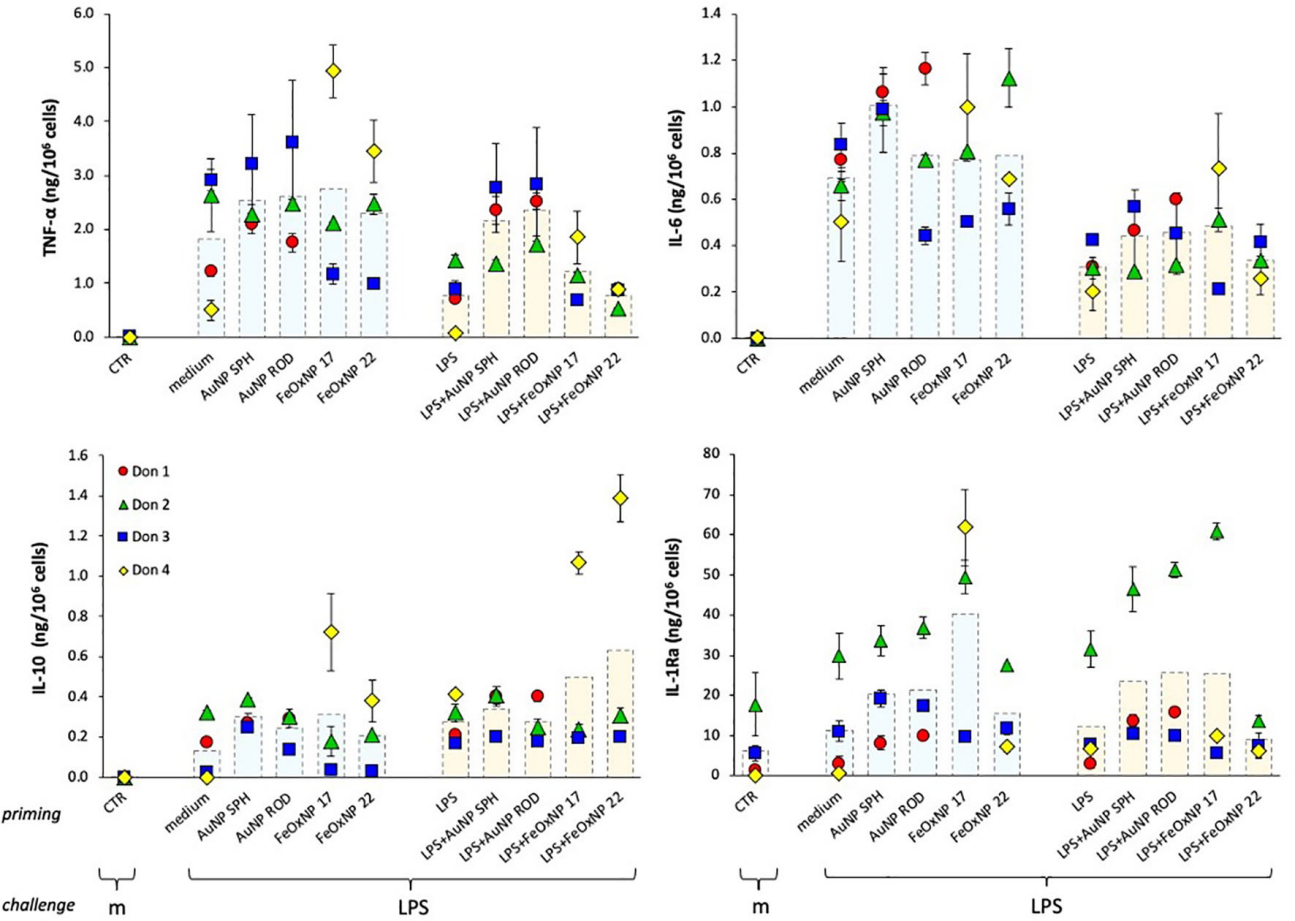
Innate immune memory response of human monocytes primed with NP alone or together with LPS. Human monocytes isolated from blood of four individual donors (red, green, blue, and yellow symbols) were cultured for 24 h in culture medium alone or containing serum-precoated NP: 5.7 µg/ml AuNP SPH, 1.4 µg/ml AuNP ROD, 2 µg/ml FeOxNP 17 and 2.7 µg/ml FeOxNP 22 alone (light blue columns) or together with 1 ng/ml LPS (orange columns) (line *priming in* abscissa). Cells were then washed and rested for 6 days in the absence of stimuli, then challenged for 24 h in fresh medium alone or containing 5 ng/mL LPS (m and LPS in the abscissa line *challenge*). The production of TNFα (upper left), IL-6 (upper right), IL-10 (lower left) and IL-1Ra (lower right) was measured in the 24 h supernatants by ELISA. Data are presented as individual donors’ values (coloured symbols) ± SD and as mean of the individual values (dotted columns). The basal response of control cells (CTR), *i.e.*, primed or unprimed cells rested for 6 days and then exposed for 24 h to medium alone, is shown at the extreme left in each panel (empty columns).

As for the primary response, the memory response was assessed in terms of production of inflammatory and anti-inflammatory cytokines, and the results are reported in **Figure 3**. Similar to the primary response, LPS challenge of medium-primed cells showed a general induction of TNFα, IL-6 and IL-10 production, and a small increase over the substantial baseline production of IL-1Ra (compare the columns “CTR”, no challenge, and “medium”, medium-primed challenged with LPS). It is interesting to note that cells primed with LPS responded to challenge with a lower production of the inflammatory cytokines TNFα and IL-6 in all donors, while there was no change in the production of the anti-inflammatory factors relative to the response of medium-primed cells (**Figure 3**, orange column “LPS” *vs*. light blue column “medium” in the priming line). This is in line with the features of the “endotoxin tolerance” that implies a reduced inflammatory response upon repeated exposures in order to avoid a destructive inflammatory reaction to a recurrent challenge. Priming with NP showed variable effects, which seem to depend more on the donor than on the NP chemistry, size or shape. The innate memory induced by co-priming with LPS and NP generally shows distinct features from those induced by either agent alone. In the case of TNFα production, priming with LPS + AuNP abolished the tolerance observed in LPS-primed cells and reconducted the response to the levels shown by unprimed cells in 2/3 donors. Conversely, priming with LPS + FeOxNP showed variable, donor-dependent and NP size-dependent effects. The LPS-induced decrease of IL-6 production was differentially affected by different NP in different donors. Likewise, the memory production of the two anti-inflammatory cytokines IL-10 and IL-1Ra showed a substantial donor-to-donor variability that did not allow for a global evaluation.

**Figure 4:**
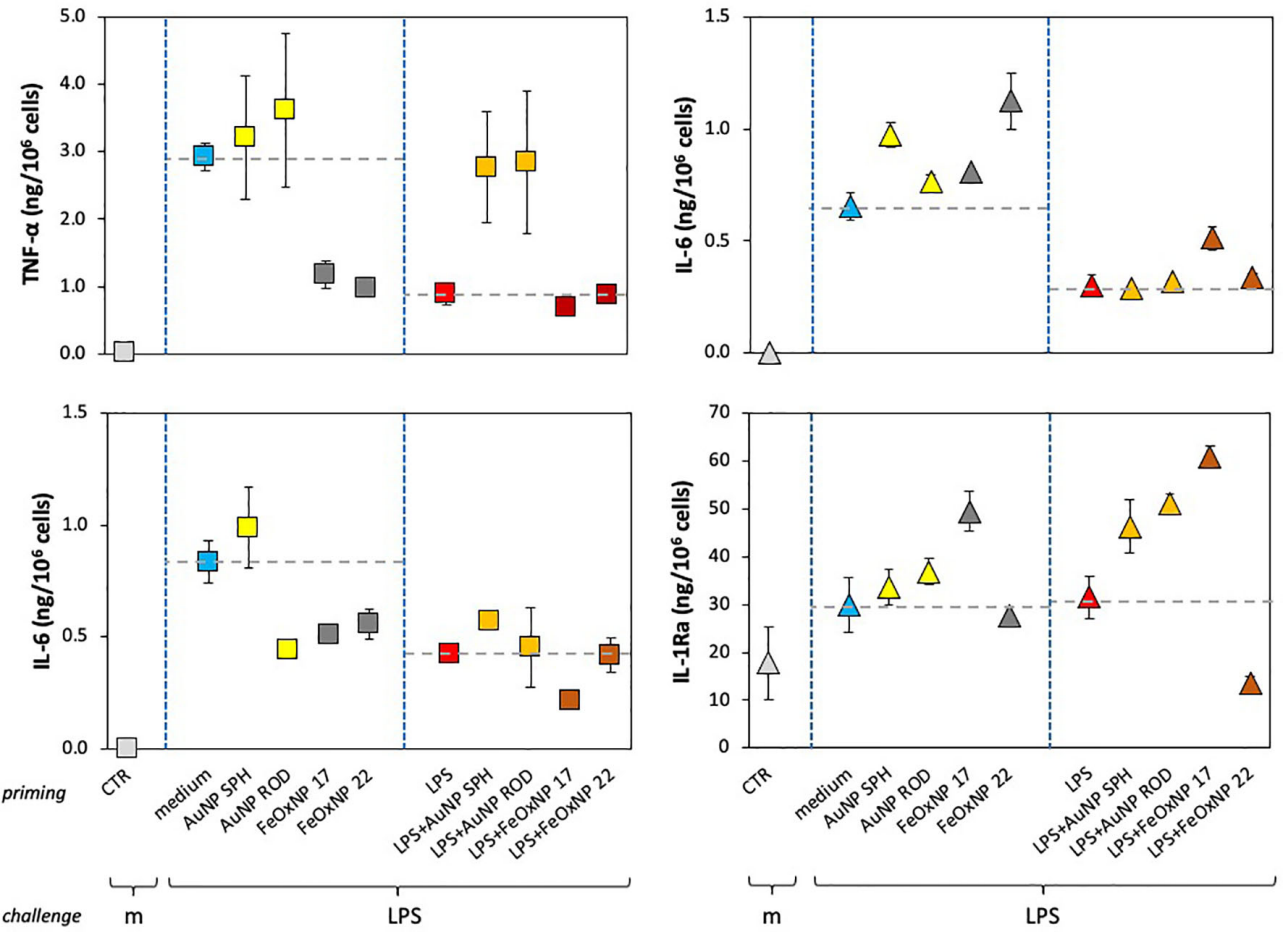
Innate memory response of human monocytes from individual donors, primed with AuNP or FeOxNP alone or together with LPS. Human monocytes isolated from blood of two individual donors (donor 3, square symbols, left panels; donor 2, triangle symbols, right panels) were cultured for 24 h in culture medium alone or containing serum-precoated NP: 5.7 µg/ml AuNP SPH, 1.4 µg/ml AuNP ROD, 2 µg/ml FeOxNP 17 and 2.7 µg/ml FeOxNP 22, alone or together with 1 ng/ml LPS (line *priming in* abscissa). Cells were then washed and rested for 6 days in the absence of stimuli, then challenged for 24 h in fresh medium alone or containing 5 ng/mL LPS (m and LPS in the abscissa line *challenge*). The production of TNFα (upper left), IL-6 (upper right and lower left), and IL-1Ra (lower right) was measured in the 24 h supernatants by ELISA. Data are reported as mean ± SD of replicate determination from individual donors out of four tested (all shown in **Figure 3**). Horizontal dotted lines represent the reference values of cells primed with medium alone and cells primed with LPS in the two parts of each panel. The basal response of control cells (CTR), *i.e.*, primed or unprimed cells rested for 6 days and then exposed for 24 h to medium alone, is shown at the extreme left in each panel (grey symbols). Statistical significance is as follows. Upper left panel: CTR *vs*. medium, *p <*0.0001; medium *vs.* LPS, *p <*0.005; medium *vs*. FeOxNP, *p <*0.005; LPS *vs*. LPS + AuNP, *p <*0.05. Upper right: CTR *vs*. medium, *p <*0.0001; medium *vs.* LPS, *p <*0.005; medium *vs*. AuNP SPH and FeOxNP 22, *p <*0.05; LPS vs. LPS + FeOxNP 17, *p <*0.05. Lower left: CTR *vs*. medium, *p <*0.0001; medium *vs.* LPS, *p <*0.01; medium *vs*. AuNP ROD, FeOxNP 17 and FeOxNP 22, *p <*0.05; LPS *vs*. LPS + FeOxNP 17, *p <*0.05. Lower right: medium *vs*. FeOxNP 17, *p <*0.05; LPS *vs*. LPS + all NP, *p <*0.05. All other relevant comparisons are not significant.

**Figure 5:**
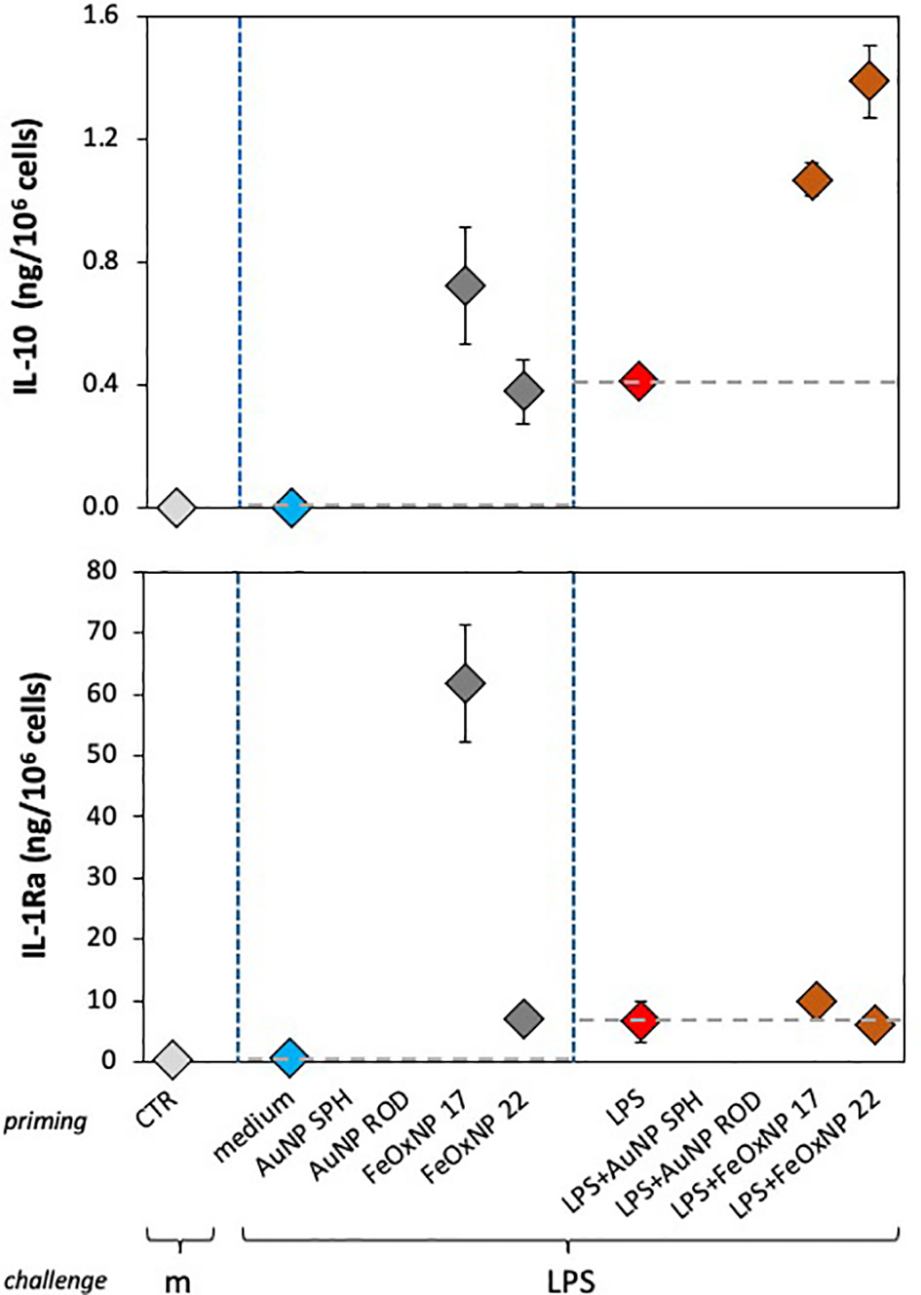
Anti-inflammatory innate memory response of human monocytes from one individual donor, primed with FeOxNP alone or together with LPS. Human monocytes isolated from blood of one individual donor (donor 4, diamond symbols); were cultured for 24 h in culture medium alone or containing serum-precoated FeOxNP (2 µg/ml FeOxNP 17 and 2.7 µg/ml FeOxNP 22) alone or together with 1 ng/ml LPS (line *priming in* abscissa). Cells were then washed and rested for 6 days in the absence of stimuli, then challenged for 24 h in fresh medium alone or containing 5 ng/mL LPS (m and LPS in the abscissa line *challenge*). The production of IL-10 (upper panel) and IL-1Ra (lower panel) was measured in the 24 h supernatants by ELISA (complete data shown in **Figure 3**). Horizontal dotted lines represent the reference values of cells primed with medium alone and cells primed with LPS in the two parts of each panel. The basal response of control cells (CTR), *i.e.*, primed or unprimed cells rested for 6 days and then exposed for 24 h to medium alone, is shown at the extreme left in each panel (grey symbols). Data are reported as mean ± SD of replicate determination from individual donors out of four tested (all shown in **Figure 3**). Statistical significance is as follows. Upper panel: medium *vs.* LPS, *p <*0.05; medium *vs*. both FeOxNP, *p <*0.05; LPS *vs*. LPS + FeOxNP, *p <*0.05; FeOxNP 17 *vs*. LPS + FeOxNP 17, *p <*0.05. Lower panel: medium *vs*. FeOxNP 17, *p <*0.05; FeOxNP 17 *vs*. FeOxNP22, *p <*0.05. All other relevant comparisons are not significant.

From the data shown above, it is clear that the significant donor-to donor variability prevents drawing general conclusions. By examining the individual responses, it is evident that each donor has her/his own capacity to discriminate between different NP. The results in **Figures 4**, **5** (data taken from **Figure 3**) underline such donor-specific capacity to distinguish between NP chemistry, shape and size. **Figure 4** reports the production of an inflammatory and an anti-inflammatory cytokine in the memory response of two individual donors, donor 2 (triangles, right panels) and donor 3 (squares, left panels). The secondary (memory) production of TNFα by donor 3 showed no significant changes in cells primed with AuNP but a substantial decrease in cells primed with FeOxNP. In addition, the decrease of TNFα in LPS-primed cells (a typical “tolerance” type memory response) was abolished if cells were co-primed with AuNP, while co-priming with FeOxNP had no effect (**Figure 4**, upper left panel). Thus, it seems that cells from donor 3 “see” the difference in the NP chemical composition but do not distinguish between shapes and size, as spherical and rod-shaped AuNP have comparable effects, and FeOxNP of both sizes equally decrease the TNFα production at challenge. However, when examining the NP effects on the IL-6 memory response, it becomes clear that cells from donor 3 also recognise size and shape. The memory production of IL-6 in cells primed with AuNP ROD was significantly decreased, while priming with AuNP SPH had no effect. Also, co-priming with LPS and FeOxNP shows that while FeOxNP 22 have no effect, co-priming with FeOxNP 17 substantially decreased IL-6 production (**Figure 4**, lower left panel).

When examining the memory responses of donor 2 (**Figure 4**, right panels), we can see a different picture. Priming with AuNP SPH increased the memory production of inflammatory IL-6 (upper right panel), while AuNP ROD were ineffective; FeOxNP 17 were likewise ineffective, while FeOxNP 22 increased the response. This suggests that donor 2 can distinguish between shapes and sizes. Interestingly, none of the NP had a significant effect on the tolerance type of memory induced by LPS priming, except for FeOxNP 17, which partially reverted tolerance. The memory effects on the anti-inflammatory factor IL-1Ra are very different: only priming with FeOxNP 17 increased the secondary production of IL-1Ra, while all NP interfered with the LPS priming (a priming that *per se* did not significantly change the cytokine production). Thus, priming with LPS plus AuNP SPH, AuNP ROD or FeOxNP17 increased the secondary production of IL-1Ra, while co-priming with FeOxNP 22 decreased it. Therefore, donor 2 seems able to discriminate between both size and shape.

In the present study, four cytokines were evaluated, two with inflammatory activity and two with anti-inflammatory effects. The data in **Figure 4** compare, in two individual donors, two inflammatory factors (TNFα and IL-6 for donor 3) and an inflammatory factor with an anti-inflammatory cytokine (IL-6 and IL-1Ra for donor 2), as example of the different capacity of different donors to recognise the NP chemistry, size and shape. In **Figure 5** we show that also between two anti-inflammatory factors the NP-induced memory can be different. Control medium-primed cells of donor 4 produced essentially undetectable levels of both anti-inflammatory factors IL-10 (upper panel) and IL-1Ra (lower panel) in response to an LPS challenge. If cells were primed with FeOxNP (no AuNP priming was included in this experiment), the production of IL-10 was significant and comparable between the two NP sizes, while the production of IL-1Ra was significantly higher in FeOxNP 17- *vs*. FeOxNP 22-primed cells. In LPS-primed cells, co-priming with both FeOxNP types increased IL-10 production, while no effect was detectable for IL-1Ra. Thus, the strong anti-inflammatory effect of FeOxNP 17 priming (increase in IL-10 and strong increase in IL-1Ra) was partially abolished if priming occurred in the presence of LPS, as priming with LPS + FeOxNP 17 induced similar IL-10 and much lower IL-1Ra levels than priming with FeOxNP 17 alone. Conversely, the more limited anti-inflammatory effect of FeOxNP 22 (a small induction of both IL-10 and IL-1Ra) was increased in LPS co-primed cells (increase in IL-10, no change in IL-1Ra).

## Conclusions

This study confirms previous observations that engineered NP can modulate human innate memory (62–66). A direct capacity of inducing memory, thereby changing the secondary response of human monocytes to a microbial challenge, was observed with AuNP of two different shapes (both coated with citrate and serum proteins), and with iron oxide NP of two different sizes (both coated with the sulfobetaine ZDS and pre-treated with serum). In addition, all four NP types were able to modulate the memory response induced by LPS. Our data also show that memory can be differently induced depending on the NP shape (in cells from the same donor the memory profile induced by AuNP SPH is different from that induced by AuNP ROD) and on the NP size (in the same donor the memory profile induced by FeOxNP 17 is different from that induced by FeOxNP 22). Dependence on the surface chemistry is more difficult to ascertain since in our NP different coating is associated with different composition, size and shape of the inorganic core. However, when the response difference between the AuNP pair and the FeOx NP pair is much larger than those within the pairs, as occurs for the TNFα memory effect, the main causal factor most likely is the surface chemistry. Overall, the most important observation is that the memory effects are strongly donor-dependent, thereby preventing a generalisation of the NP effects.

We examined the global scenario of NP-generated memory in this study by simplifying the memory responses to NP as increase of inflammation/immunostimulation (+), no change (=) and decrease (−) *vs*. the response of control cells primed in the absence of NP. The results are shown in **Table 3**. Since for every NP we have tested the memory effects on the production of two inflammatory and two anti-inflammatory factors, we have assessed the overall memory effect in each donor by considering the balance between changes in the production of inflammatory *vs*. anti-inflammatory factors, in order to obtain an individual profile of the NP-induced memory responses. The summary in **Table 4** depicts the NP-specific donor-specific memory profiles based on the data summarised in **Table 3**. The general finding is that each donor reacts differently to the same NP. For instance, memory induced by priming with AuNP SPH induces no overall change in the response of donor 1 (the lack of change being due to the increase of two inflammatory markers balanced by the increase in two anti-inflammatory markers), a more inflammatory response in donor 2 and a more anti-inflammatory response in donor 3. Strikingly, the memory profile induced by AuNP SPH together with LPS is different from that induced by NP alone, being more anti-inflammatory for donor 1 (*vs.* no change with NP alone), and opposite for donor 2 (more anti-inflammatory) and donor 3 (more inflammatory).

**Table 3:** Changes in cytokine production in NP-induced innate memory responses.

Priming^1^		Cytokine^2^	Donors^3^			
LPS	NP		D1	D2	D3	D4
No	AuNP SPH	TNFα	+	=	=	*nt*
		IL-6	+	+	=	*nt*
		IL-10	+	=	+	*nt*
		IL-1Ra	+	=	=	*nt*
	AuNP ROD	TNFα	=	=	=	*nt*
		IL-6	+	=	−	*nt*
		IL-10	+	=	+	*nt*
		IL-1Ra	+	=	=	*nt*
	FeOxNP 17	TNFα	*nt*	=	−	−
		IL-6	*nt*	=	−	+
		IL-10	*nt*	=	=	+
		IL-1Ra	*nt*	+	=	+
	FeOxNP 22	TNFα	*nt*	=	−	+
		IL-6	*nt*	+	−	=
		IL-10	*nt*	=	=	+
		IL-1Ra	*nt*	=	=	+
Yes	AuNP SPH	TNFα	+	=	+	*nt*
		IL-6	=	=	=	*nt*
		IL-10	+	=	=	*nt*
		IL-1Ra	+	+	=	*nt*
	AuNP ROD	TNFα	+	=	+	*nt*
		IL-6	+	=	=	*nt*
		IL-10	+	=	=	*nt*
		IL-1Ra	+	+	=	*nt*
	FeOxNP 17	TNFα	*nt*	=	=	−
		IL-6	*nt*	+	−	+
		IL-10	*nt*	=	=	+
		IL-1Ra	*nt*	+	=	=
	FeOxNP 22	TNFα	*nt*	−	=	−
		IL-6	*nt*	=	=	=
		IL-10	*nt*	=	=	+
		IL-1Ra	*nt*	−	=	=

^1^ Human monocytes were primed in vitro with NP alone or in the presence of 1 ng/mL LPS for 24 h, then washed, rested for 6 days and eventually challenged for 24 h with 5 ng/mL LPS.

^2^ The two inflammatory cytokines TNFα and IL-6, and the two anti-inflammatory cytokines IL-10 and IL-1Ra were measured in the 24 h supernatant of LPS-challenged monocytes.

^3^ The NP effects on memory responses were assessed in four individual donors, indicated as D1, D2, D3 and D4, and expressed as statistically significant changes (increase, +; decrease, −; no change, =) vs. control cells (medium-primed or LPS-primed). Actual values are shown in **Figure 3**.

nt, not tested.

**Table 4:** Individual profiling of the NP-induced innate memory responses.

NP^1^	Donor	Memory profiling^2^ upon priming with^3^			
		NP		LPS + NP	
AuNP SPH	D1	2+/2−	Balance	1+/2−/1=	Towards Tolerance
	D2	1+/3=	Towards Potentiation	1−/3=	Towards Tolerance
	D3	1−/3=	Towards Tolerance	1+/3=	Towards Potentiation
AuNP ROD	D1	1+/2−/1=	Towards Tolerance	2+/2−	Balance
	D2	4=	Balance	1−/3=	Towards Tolerance
	D3	2−/2=	Tolerance	1+/3=	Towards Potentiation
FeOxNP 17	D2	1−/3=	Towards Tolerance	1+/1−/2=	Balance
	D3	2−/2=	Tolerance	1−/3=	Towards Tolerance
	D4	1+/3−	Tolerance	1+/2−/1=	Towards Tolerance
FeOxNP 22	D2	1+/3=	Towards Potentiation	1+/1−/2=	Balance
	D3	2−/2=	Tolerance	4=	Balance
	D4	1+/2−/1=	Towards Tolerance	2−/2=	Tolerance

^1^The four types of NP used for priming (memory induction) are indicated.

^2^The overall NP effects on memory responses are described as potentiation (i.e., increased inflammation/immunostimulation) or tolerance (i.e., increased anti-inflammation/immunosuppression) or balance, based on the changes in the production of inflammation-related cytokines in comparison to control cells (all listed in **Table 3**). For the individual memory profiling, four cytokines were assessed in cells from each donor and their NP-induced memory changes indicated as + (when observing an increase in the production of the inflammatory factors TNFα and IL-6, or a decrease in the production of the anti-inflammatory cytokines IL-10 and IL-1Ra), − (when observing a decrease in the production of the inflammatory factors TNFα and IL-6, or an increase in the production of the anti-inflammatory cytokines IL-10 and IL-1Ra) or = (no change in the cytokine production). Cytokines were measured in the 24 h supernatant of LPS-challenged monocytes. Potentiation and tolerance were considered clear when at least 2/4 parameters were changed in the same direction, and as a tendency when only 1/4 parameters was changed.

^3^The memory responses were assessed in cells primed with NP alone or NP and LPS and all challenged with LPS.

Memory-dependent changes are detected in most cases, both in the direction of an enhanced secondary response (potentiation) and in the direction of a reduced secondary response (tolerance). In general, however, these changes do not seem substantial, as they are based on the change of one parameter out of four measured. Thus, we have defined such partial changes as “towards potentiation” and “towards tolerance”, to define a tendency rather than a full change of reactivity. Only in few cases we have observed a clear tolerance (change in at least 2/4 parameters), while we could never detect a clear potentiation. This may be interpreted as a general inability of NP to induce a potentiation of subsequent reactivity, *i.e.*, we should not expect excessive inflammation upon subsequent challenges. On the other hand, there is the possibility, in some donors with some NP types, of a lower/inadequate secondary response, which might imply higher susceptibility to infections/diseases. Interestingly, in several cases we can observe a similar overall response between donors, as for instance in cells from donors 2 and 3 primed with FeOxNP 22 together with LPS, which showed an unchanged overall response. However, there is a difference between the two donors for attaining the same overall effects, with donor 3 showing no change in the four parameters, while donor 2 showed a decrease in one inflammatory parameter (TNFα) counterbalanced by a decrease in one anti-inflammatory parameter (IL-1Ra).

As general conclusion, we have observed a strongly individual memory response of human innate cells primed with NP, to confirm previous indications (62, 64, 66). The NP-induced memory response can differ between donors for the same type of NP, a finding that supports the need for a personalised memory profiling, before administration of nanoimaging materials for diagnostic scopes. This would allow us to predict the possible impact of NP on the capacity of the patient to adequately react to future challenges, thereby allowing for selecting patients unlikely to develop innate memory alterations and avoiding treating those at risk of developing health-impairing reactivities. In the case of both nanoimaging and nanotherapeutic materials, a preventive innate memory profiling of the patient will contribute to implementing a better targeted treatment and a safer patient management.

## Data Availability Statement

The original contributions presented in the study are included in the article/**Supplementary Material**. Further inquiries can be directed to the corresponding authors.

## Ethics Statement

The studies involving human participants were reviewed and approved by Regional Ethics Committee for Clinical Experimentation of the Tuscany Region (Ethics Committee Register n. 14,914 of May 16, 2019). Written informed consent for participation was not required for this study in accordance with the national legislation and the institutional requirements.

## Author Contributions

GDC, AF, RLS and AP synthesized and characterised the nanomaterials. GDC, MM, YL, AC, TH, BJS and GS contributed to the experimental work. DB and PI planned the study. DB wrote the manuscript. AP and MM contributed to writing the manuscript. GDC, AP, DB and PI prepared the figures. PI performed the statistical analysis. All authors contributed to the article and approved the submitted version.

## Funding

This work was supported by the EU Commission H2020 projects ENDONANO (GA 812661) and PANDORA (GA 671881), the FP7 project HUMUNITY (GA 316383), the Italian MIUR InterOmics Flagship projects MEMORAT and MAME, and the Italian MIUR/PRIN-20173ZECCM. This work was partially carried out in the frame of the JRC Visiting Scientist agreement no. 05/JRC.F.2/2019 (Directorate F - Health, Consumers and Reference Materials, Consumer Products Safety, Nanobiotechnology Lab).

## Conflict of Interest

The authors declare that the research was conducted in the absence of any commercial or financial relationships that could be construed as a potential conflict of interest.

## Publisher’s Note

All claims expressed in this article are solely those of the authors and do not necessarily represent those of their affiliated organizations, or those of the publisher, the editors and the reviewers. Any product that may be evaluated in this article, or claim that may be made by its manufacturer, is not guaranteed or endorsed by the publisher.

## Supplementary Material

Supplementary Figure 1 TEM (number-weighted) NP size distribution. Histograms representing the size distribution from TEM images. **(A)** AuNP SPH; **(B)** AuNP ROD (the distribution of both length and width are shown in the left and right panels, respectively); **(C)** FeOxNP 17; and d) FeOxNP 22. The red line in **(C, D)** is the best-fit log-normal distribution. The size data are reported in the inserts and in **Table 1**.

Supplementary Figure 2 DLS intensity-weighted distribution of the hydrodynamic diameter of AuNP SPH. Intensity-weighted distribution (*P_I_*) of the hydrodynamic diameter of AuNP SPH in WFI (black), PBS (blue) and RPMI-1640 after pre-treatment with human serum (red). Error bars are shown. The largest PI peak is centered at 16 nm for AuNP SPH in WFI, 15 nm for AuNP SPH in PBS, and 21 nm for AuNP SPH in RPMI-1640 after pre-treatment with human serum.

Supplementary Figure 3 AuNP uptake by monocytes. TEM images of AuNP SPH internalized by monocytes and associated within vesicles. Size bars are 1 mm in the left panel and 500 nm in the right panel.
