# Supplementary figures and images for: Personalised Profiling of Innate Immune Memory Induced by Nano-Imaging Particles in Human Monocytes
Source: Front Immunol. 2021 Aug 6;12:692165. doi: 10.3389/fimmu.2021.692165 (PMC8377278; doi:10.3389/fimmu.2021.692165)

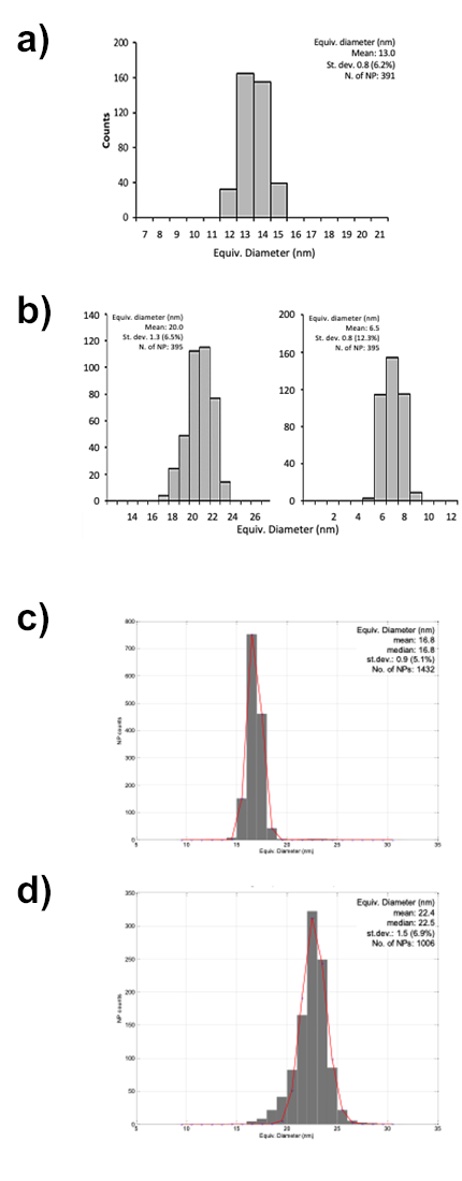

Supplement: Supplementary Figure 1 — TEM (number-weighted) NP size distribution. Histograms representing the size distribution from TEM images. (A) AuNP SPH; (B) AuNP ROD (the distribution of both length and width are shown in the left and right panels, respectively); (C) FeOxNP 17; and d) FeOxNP 22. The red line in (C, D) is the best-fit log-normal distribution. The size data are reported in the inserts and in Table 1. [file Image_1.jpeg]

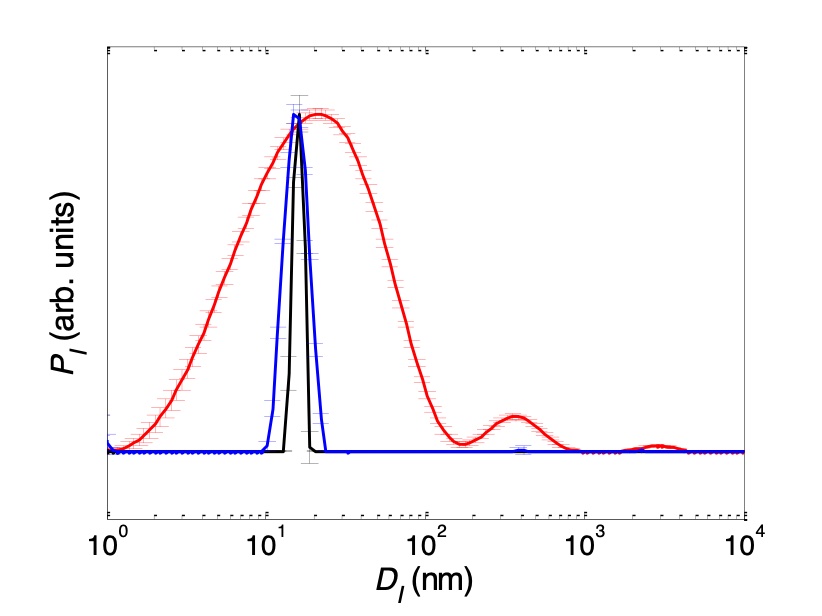

Supplement: Supplementary Figure 2 — DLS intensity-weighted distribution of the hydrodynamic diameter of AuNP SPH. Intensity-weighted distribution (PI) of the hydrodynamic diameter of AuNP SPH in WFI (black), PBS (blue) and RPMI-1640 after pre-treatment with human serum (red). Error bars are shown. The largest PI peak is centered at 16 nm for AuNP SPH in WFI, 15 nm for AuNP SPH in PBS, and 21 nm for AuNP SPH in RPMI-1640 after pre-treatment with human serum. [file Image_2.jpeg]

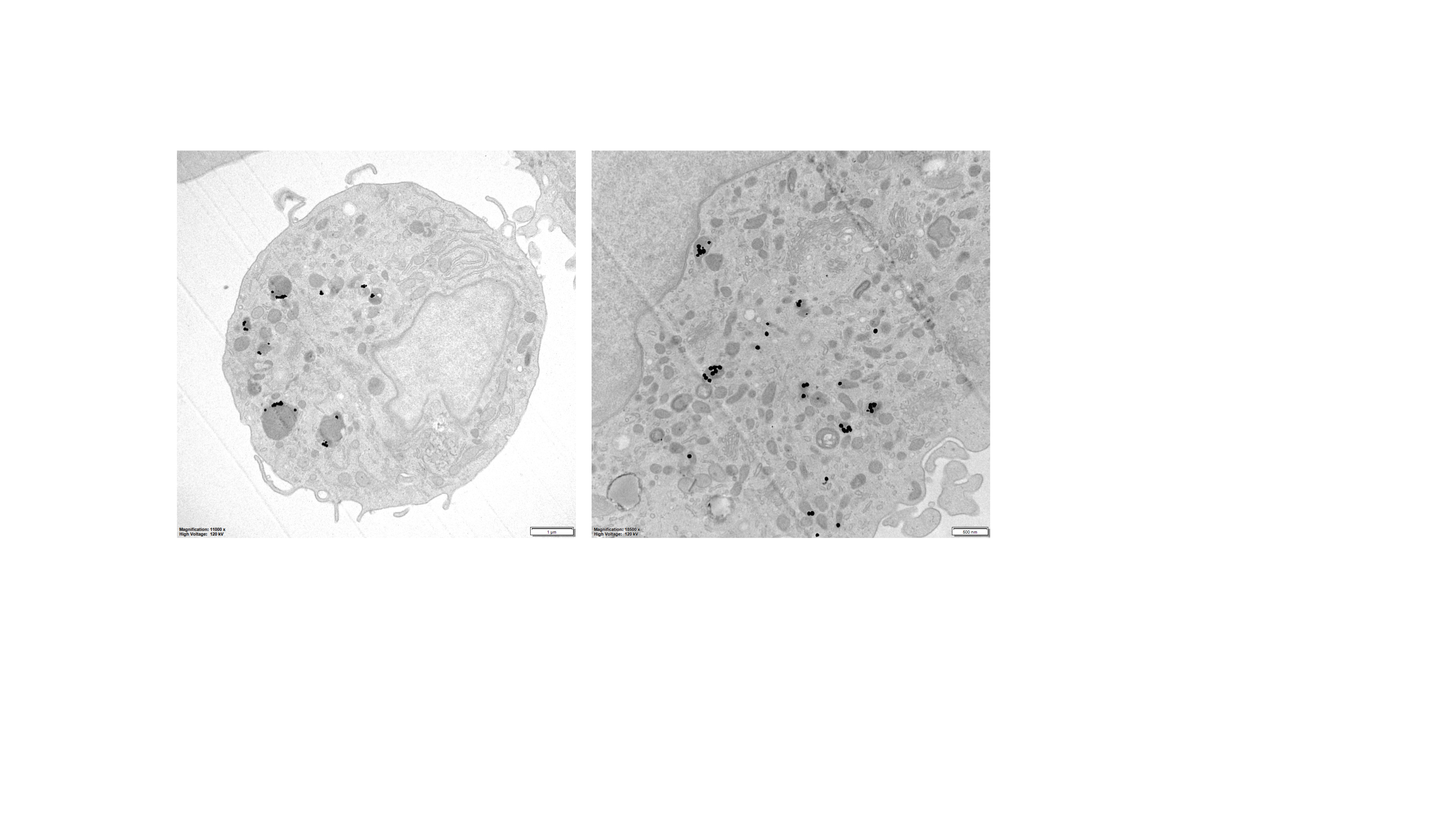

Supplement: Supplementary Figure 3 — AuNP uptake by monocytes. TEM images of AuNP SPH internalized by monocytes and associated within vesicles. Size bars are 1 mm in the left panel and 500 nm in the right panel. [file Image_3.tiff]
